# Investigation of optical force on magnetic nanoparticles with magnetic-fluid-filled Fabry-Perot interferometer

**DOI:** 10.1038/s41598-018-30092-7

**Published:** 2018-08-17

**Authors:** Tianjun Yao, Shengli Pu, Jie Rao, Jianming Zhang

**Affiliations:** 1 College of Science, University of Shanghai for Science and Technology, Shanghai, 200093 China; 2 Shanghai Key Laboratory of Modern Optical System, University of Shanghai for Science and Technology, Shanghai, 200093 China

## Abstract

The optical force acting on the magnetic nanoparticles (MNPs) is investigated with the magnetic-fluid-filled fiber-optic Fabry-Perot interferometer. The shift of interference spectra is related with the local refractive index variation in the light path, which is assigned to the optical-force-induced outward movement of MNPs. The influence of magnetic fluid’s viscosity, ambient temperature, strength and orientation of the externally applied magnetic field on the optical-force-induced MNPs’ movement is studied in details. The results of this work provide a further understanding of interaction between light and MNPs and clarify the dynamic micro-processes of MNPs within magnetic fluid under external stimuli. It may have the potentials in the fields of light-controllable magnetic-fluid-based devices and vector magnetic field detection.

## Introduction

In 1970, Ashkin first observed that the transparent dielectric particles can be trapped within the beam center, which is assigned to the optical forces^1^. The technology based on optical forces is now called optical tweezers. As optical tweezers can efficiently trap and manipulate various particles (such as atoms^2^, quantum dots^3^, metal nanospheres^4^, microscopic bubbles^5^, and cells^6^), they have been intensively used in various scientific researches^7–10^. Optical force is also called electromagnetic radiation force, which is rooted in electromagnetic field action on medium. When electromagnetic field impinges on medium, the response of dielectric property and magnetization characteristic of the medium to the electromagnetic field leads to the behavior of forces and momentum^11–13^. Generally, optical forces can be decomposed into gradient and scattering-plus-absorption force^1,7,14,15^. Depending on physical properties (such as dielectric properties, magnetization characteristic) of the particles and the environment^1,11,14^, the optical forces on particles can be attractive or repulsive^15–18^.

Magnetic fluid (MF) is a kind of stable magnetic colloid consisting of surfactant-coated single-domain magnetic nanoparticles dispersed in a suitable liquid carrier^19–21^. Recently, the unique optical properties of MF, such as magnetic-field-dependent refractive index (RI)^22,23^, nonlinear properties^24^, and thermo-optic effect^25–27^, have been intensively studied due to their novel and promising applications in sensing^28–30^, photonics and detection^31–37^. RI is one of the most critical parameters for optical design and applications. It has been well studied that the RI of MF depends on the externally applied magnetic field and ambient temperature, which is attributed to the microstructural formation of magnetic nanoparticles (MNPs) within the MF^23,26^. However, for optical applications based on MF, the optical forces may “parasitically” occur and then influence the movement of MNPs, which will lead to the RI change and then affect the device performance. This has not been revealed in the past studies.

To this end, two kinds of MF-filled fiber-optic Fabry-Perot interferometer (MF-FPI) are fabricated. Their optical response to incident light is investigated. The optical force on the MNPs is found to be repulsive. The repulsive-optical-force-induced decrease of local MNP concentration is observed. The influence of MF’s viscosity, ambient temperature and external magnetic field on the optical force is investigated in details. The results of this work may be helpful for designing the MF-based photonic devices and extending their corresponding pragmatic applications.

## Fabrication and operating principle

**Figure 1:**
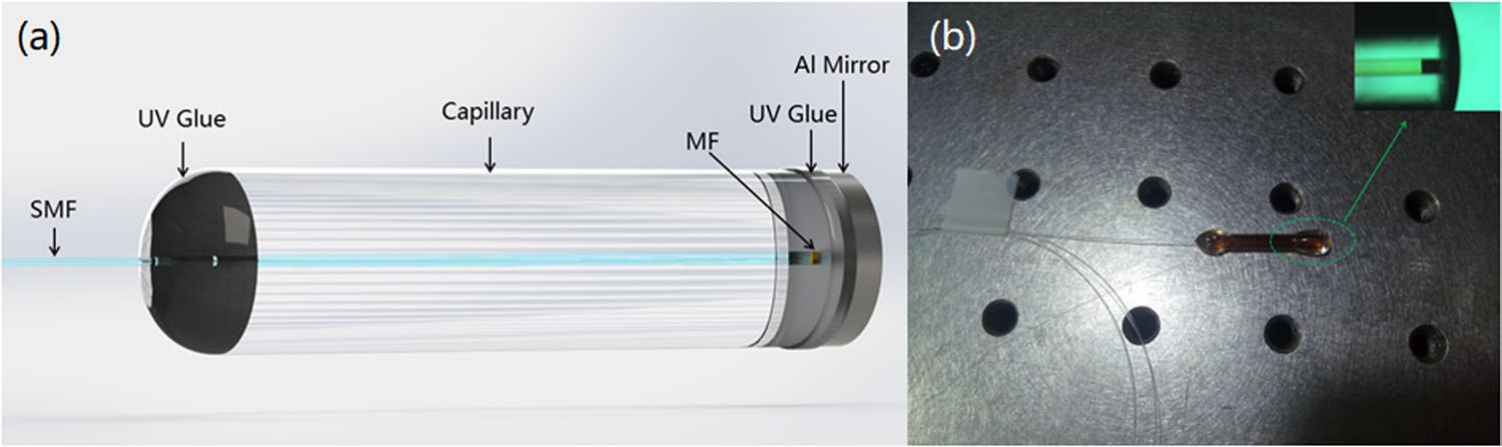
Schematic of the MF-filled Fabry-Perot interferometer (**a**) and the corresponding as-fabricated device (**b**).

Figure 1(a) shows the schematic structure of the MF-FPI. A piece of coating-stripped single-mode fiber (SMF) with flat-cleaved end is inserted into a 20 mm-long capillary. The inner diameter of the capillary is 126 μm, which is very close to that of the coating-stripped SMF. The distance between the flat-cleaved fiber end and the other open end of the capillary is around 92.9 μm. The water-based MF with density of 1.09 g/cm^3^ is used to fill the capillary from the open end. Then, a mirror is fixed on the open end of the capillary as a reflector. The UV glue is employed to seal the MF-filled capillary. By this way, the MF-FPI with cavity length of 92.9 μm is obtained. The as-fabricated device is shown in Fig. 1(b). For comparison study, another oil-based MF-FPI with cavity length of 51.5 μm is also fabricated.

The incident light comes from the lead-in fiber end-face can be represented by a Gaussian beam with waist at the fiber end-face. Then, the optical tweezer effect is intrinsically generated in the light path^38^. Thus, the optical force may act on the MNPs and the MNPs will move and redistribute radially, which will change the MNP’s concentration and then the MF’s RI in the light path. Therefore, the interference spectrum of the MF-FPI can be influenced by the incident light^39–42^. The shift of interference spectrum (Δ*λ*_*i*_) is expressed as^43,44^

1 $${\rm{\Delta }}{\lambda }_{i}={\lambda }_{i}\frac{{\rm{\Delta }}n}{n},$$

where *n* is the RI of the cavity, *λ* is the wavelength in vacuum, *i* is the interference order number. As the optical force depends on the incident power^11,12,14^, the MF’s RI variation in the light path is also dependent of the incident power. Thus, the degree of spectral shift is related with the incident power. Through monitoring the interference spectral shift of the MF-FPI, the optical-force-induced local concentration variation of MNPs and then the effect of optical force acting on the MNPs can be investigated.

## Experimental Results and Discussion

**Figure 2:**
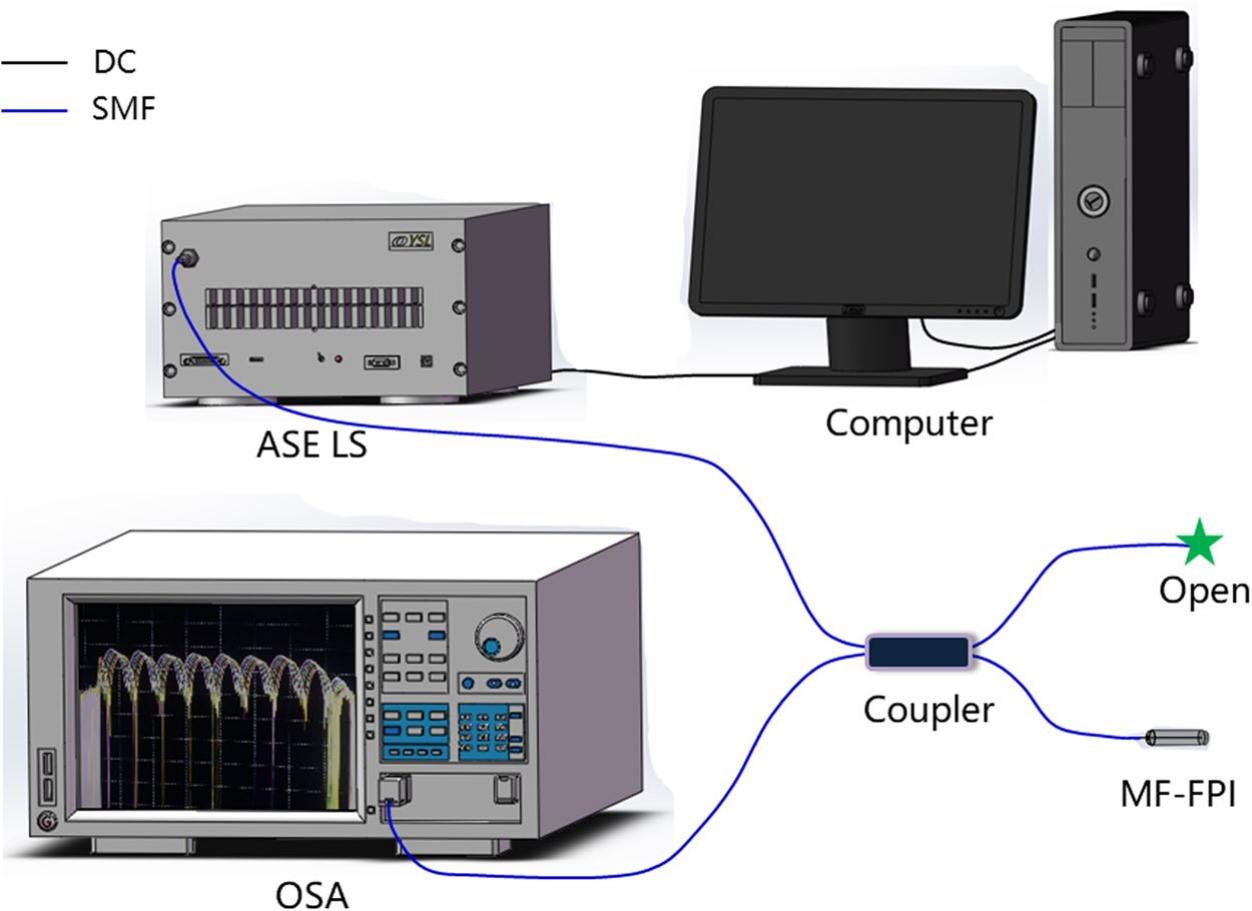
Schematic of the experimental setup for investigating the optical response properties of the MF-FPI. DC: data cable, SMF: single-mode fiber.

The schematic of the experimental setup for investigating the optical response properties of the MF-FPI structure is shown in Fig. 2. The incident light from the amplified spontaneous emission (ASE) source is led into the MF-FPI through a 3 dB coupler. The reflective spectrum of the structure is monitored and analyzed by an optical spectrum analyzer (OSA, YOKOGAWA AQ6370C). It is well-known that the absorption of MF is very strong in the visible range^27^ and decreases dramatically in the red and infrared regime^22,23^. Hence, the infrared ASE light source is utilized to reduce the possible thermal effect. The used ASE light source has a wavelength range of 1525–1610 nm and maximum output power of 10.2 mW. In order to avoid the influence of ambient temperature, the MF-FPI is put into a test tube filled with water and the constant temperature of 22.6 °C is guaranteed. Therefore, the cavity length is constant.

### Influence of incident power

**Figure 3:**
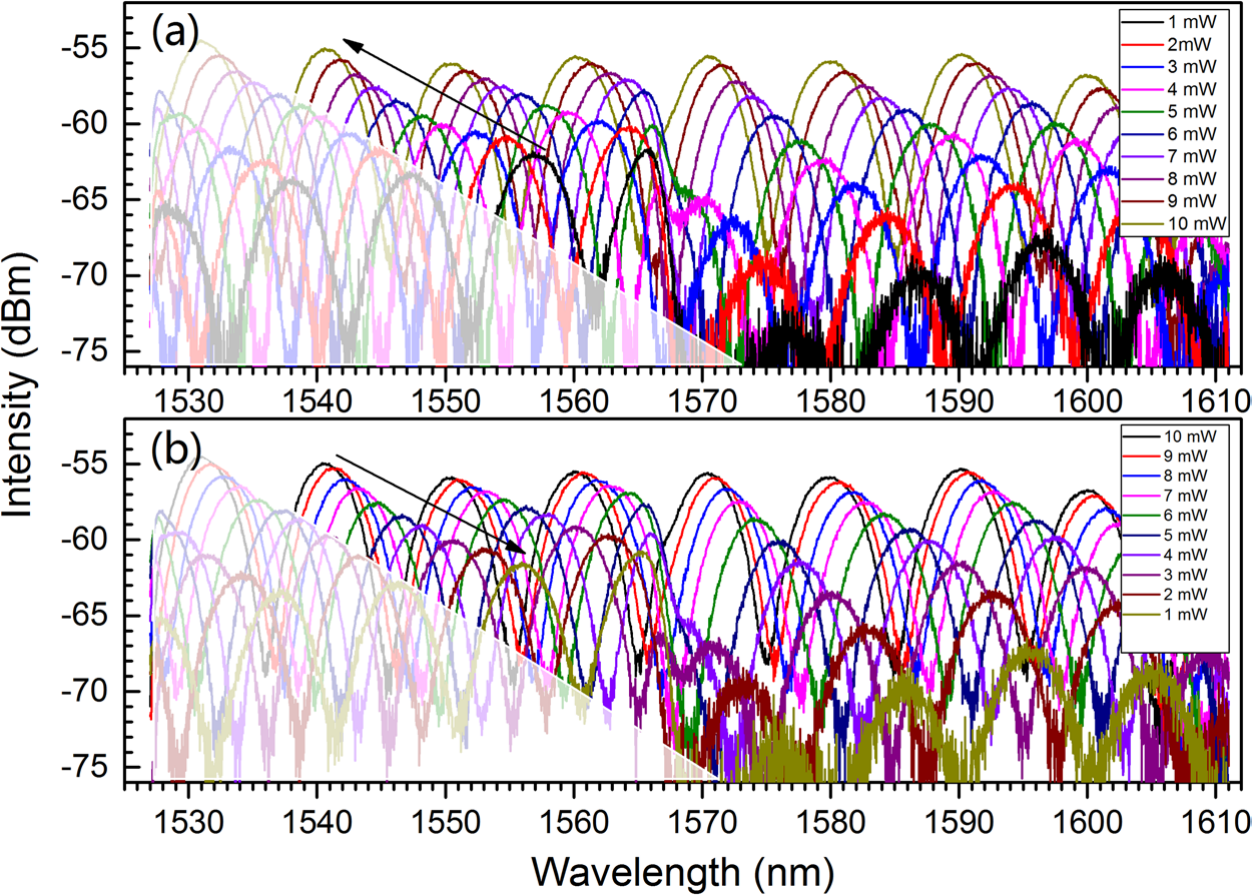
Spectra for ascending (**a**) and descending (**b**) the incident power in the range of 0.5–10 mW.

The spectra for ascending and descending the incident power in the range of 0.5–10 mW with an interval of 0.5 mW are measured. For clarity, only parts of the spectral curves are plotted in Fig. 3.

Figure 3 shows that the interference spectrum blue-shifts with the increase of incident power (see Fig. 3(a)) and shifts backward (red-shifts) when the incident power decreases (see Fig. 3(b)). This is assigned to the RI variation of MF in the light path under incident illumination, which is fundamentally related with the MNP’s concentration change derived from the action of optical force. As the RI of MF is proportional to MNP’s concentration and considering Eq. (1) as well, the blue-shift in Fig. 3(a) implies that the optical force on the MNPs is repulsive and increases with the incident power increasing. However, the red-shift in Fig. 3(b) means that the repulsive optical force decreases with the incident power decreasing. Then, the MNP’s concentration in the light path restores and the MF’s RI in the light path increases for descending incident power^45,46^. These micro-processes will be analyzed in details in the following.

**Figure 4:**
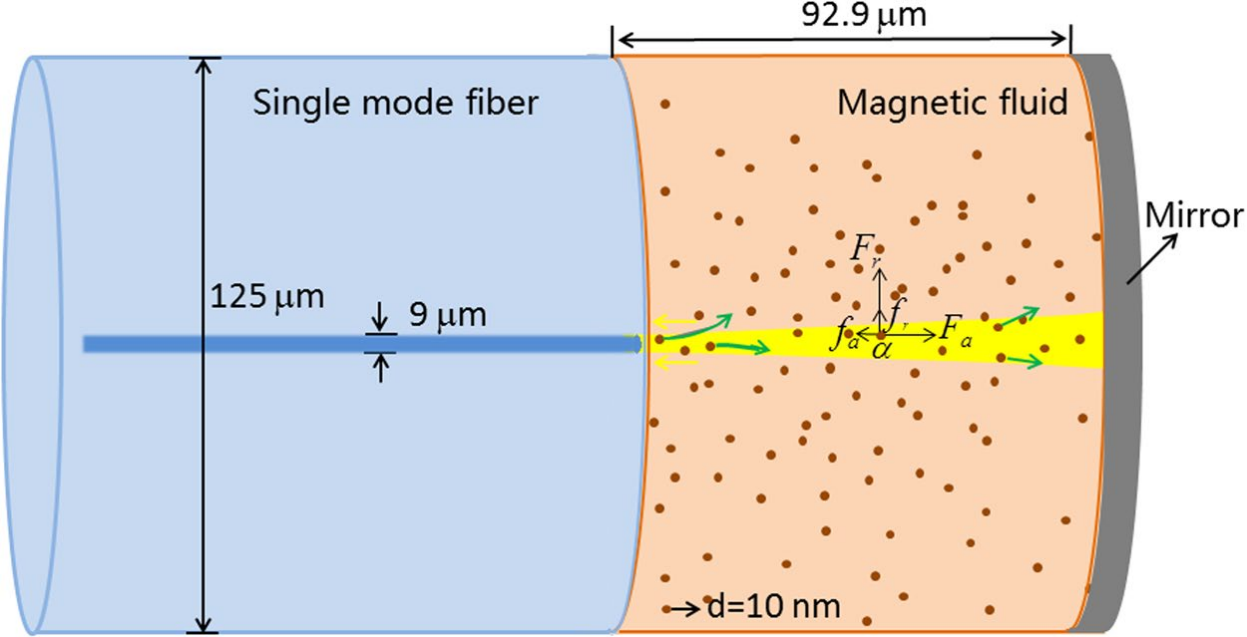
Optical force analysis for the MNP within the MF-filled interferometric structure.

Taking the *α* particle in the light path as an example (see Fig. 4), the incident-light-induced optical gradient force *F*_*r*_ acting on it is repulsive, which results in the outward movement of the *α* particle from the beam center. At the same time, *α* particle is pushed toward the direction of light propagation by the optical scattering-plus-absorption force *F*_*a*_^14,15,47^. Similarly, for the reflective-light-induced optical force acting on the *α* particle, the direction of the gradient force *f*_*r*_ is the same as that of *F*_*r*_, but the direction of the scattering-plus-absorption force *f*_*a*_ is opposite to that of *F*_*a*_. Thus, the outward movement of MNPs in the light path dominates the whole micro-process. With ascending the incident power, the repulsive optical force becomes stronger and more MNPs are pushed away from the light path. This leads to the decrease of local MF’s RI with the incident power. Thus, the interference spectrum blue-shifts with the increase of incident power. Likewise, the observed phenomena for the inverse micro-process (i.e. descending the incident power) is obvious.

**Figure 5:**
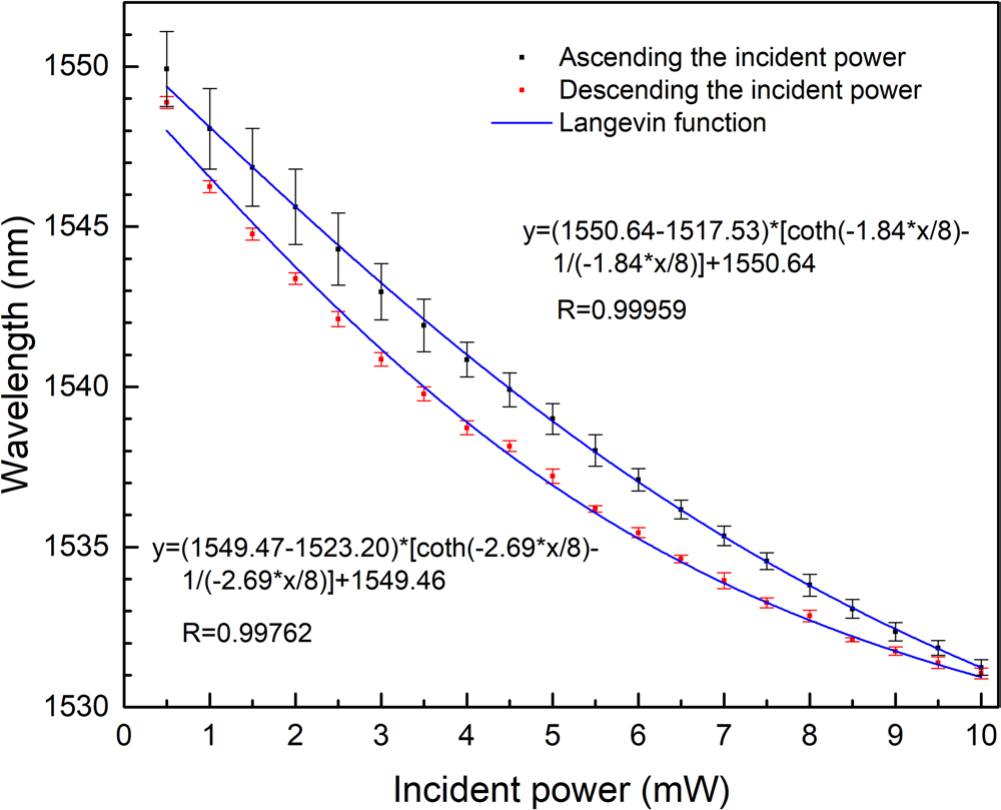
Spectral shift of peak wavelength around 1551.6 nm during the processes of increasing and decreasing incident power.

In order to investigate the spectral reversibility and repeatability of the as-fabricated structure during the processes of incident power ascending and descending, the experiments are conducted when the incident power ascends and descends four times. The corresponding average shift of the peak wavelength around 1551.6 nm with the incident power is shown in Fig. 5. Figure 5 indicates that there is a little spectral deviation between the ascending and descending processes. This may be due to the relaxation characteristics of the MNP’s concentration variation.

**Figure 6:**
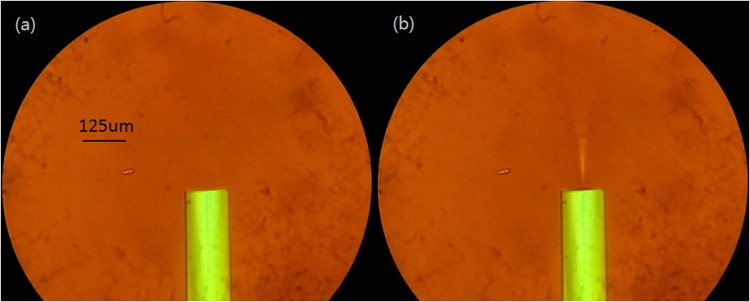
Micrographs for the SMF with flat-cleaved end immersed in water-based MF without incident light (**a**) and with 72 mW incident light (**b**).

To confirm the optical-force-driven movement of MNPs and directly observe the MNP’s concentration variation associated with the incident light, a piece of SMF with flat-cleaved end is immersed in the water-based MF. They are sandwiched between two glass slides. Tiny MNP’s concentration variation can be easily detected through the indirect interference technique, but can hardly be directly observed by the naked eye with the microscope. The higher the incident power is, the larger the variation of MNP’s concentration will be. Therefore, the high-power infrared supercontinuum light source is employed. The remarkable variation of MNP’s concentration in the light path under 72 mW incident power is observed. Figure 6 is the micrographs for the structure under zero and 72 mW incident power, respectively. The details for the variation of MNP’s concentration can be seen in the supplementary material (Video S1). Under higher incident power, more and more MNPs are repelled away from the light path, which will result in enhanced transparence in the light path as shown Fig. 6(b).

### Influence of viscosity

**Figure 7:**
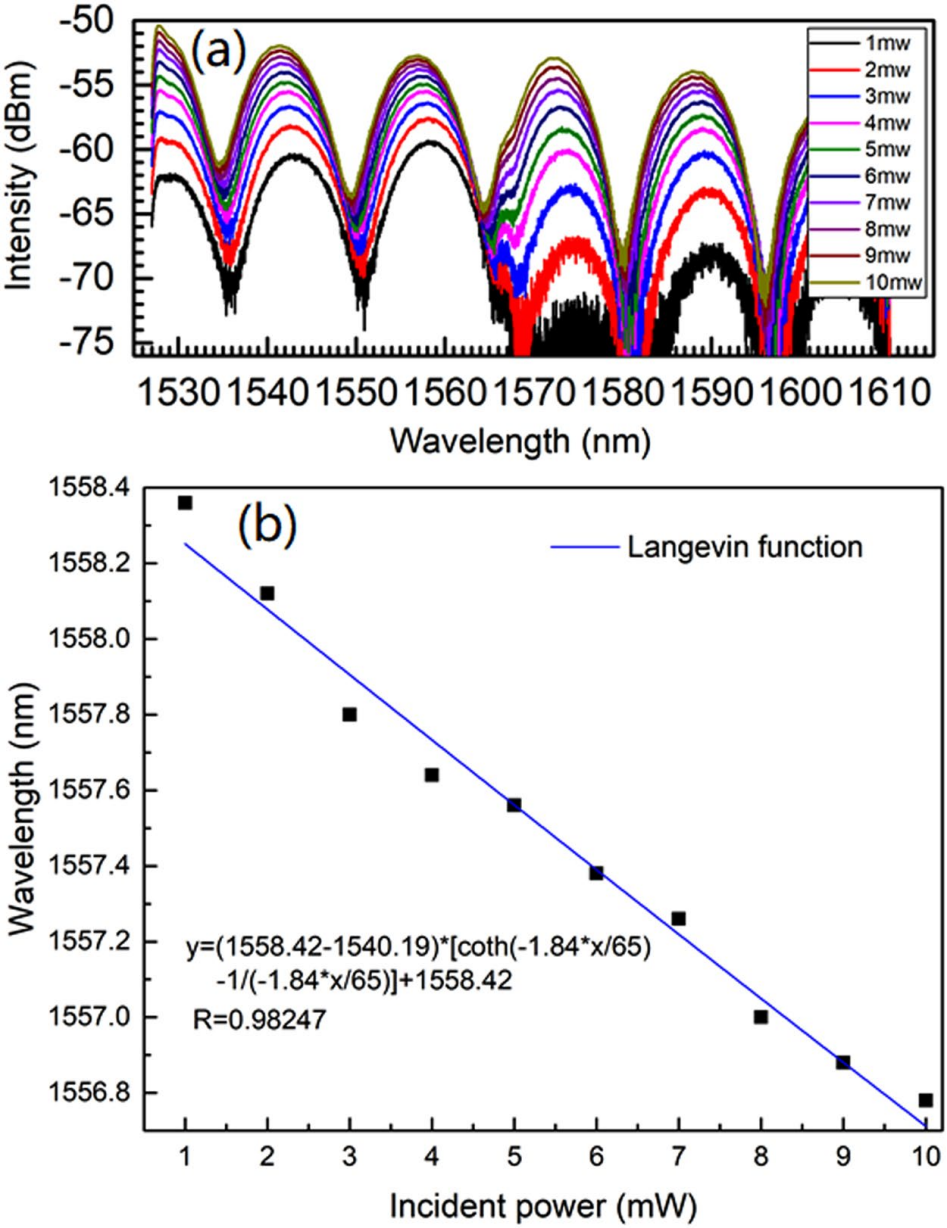
Spectra for the oil-based-MF-filled-structure under different incident powers (**a**) and shift of interference peak wavelength at around 1558.36 nm as a function of incident power (**b**).

Usually, the movement of MNPs in liquid critically depends on the viscosity^45,46,48,49^. On the other hand, the carrier liquid type of MF dominates its viscosity^21^. To investigate the influence of viscosity and then further validate the optical force effect accounting for the observed phenomena, the water-based and oil-based magnetite MFs are used for fabricating two kinds of MF-FPIs. The physical parameters of the two kinds of MF are listed in Table 1. The F-P cavity lengths of the water-based-MF-filled and oil-based-MF-filled structures are 92.9 μm and 51.5 μm, respectively. The experimental results for the water-based-MF-filled structure has been presented in Figs 3 and 5. The experimental spectra for the oil-based-MF-filled structure is shown in Fig. 7(a) and the incident-power-dependent wavelength shift is shown in Fig. 7(b). Figure 7(b) indicates that the interference peak wavelength at around 1558.36 nm has a total blue-shift of 1.58 nm. While the water-based-MF-filled structure has a total blue-shift of 16.12 nm (see Fig. 5) under the same range of incident power, which is around 10 times larger. We may note that the cavity length of the water-based MF-FPI is only ~1.8 times longer than that of the oil-based MF-FPI, which cannot account for the 10 times enhancement of spectral shift. Table 1 displays that the viscosity of the water-based MF is only around 1/8 of that of the oil-based MF. Therefore, if the optical force (viz. incident power) acting on the MNPs is the same, the optical-force-induced movement of MNPs in the light path within the water-based MF will be much easier than that within the oil-based MF. Thus, the RI variation and then the enhancement of spectral shift for the water-based MF-FPI is much greater.

**Table 1:** Physical parameters of two kinds of MF used in the experiments.

Carrier Liquid	Saturation Magnetization (Gs)	Thermal Conductivity (W∙m^−1^∙K^−1^)	Viscosity (mPa∙s, 25 °C)	Density (g/cm^3^)	Average Particle Size (nm)
Water	180	0.59	8	1.09	10
Synthetic Hydrocarbon	165	0.14	65	—	10

The aforementioned analyses imply that the RI (*n*_*MF*_) of MF within the light path decreases with the incident power (*P*), but increases with the viscosity (*η*) of carrier liquid. Usually, saturation will happen at relatively high values of the parameters, which is somewhat similar to the magnetization characteristic of MF^26^. Therefore, the Langevin-function-like *n*_*MF*_(*P*, *η*) is proposed as following

2 $${n}_{MF}(P,\,\eta )=[{n}_{0}-{n}_{s}][\coth (\sigma \frac{P}{\eta })-\frac{\eta }{\sigma P}]+{n}_{0}(P > 0),$$

where *n*_0_ is the initial RI of MF (without incident power), *n*_*s*_ is the corresponding saturated value. It is obvious that *n*_*s*_ < *n*_0_. *σ* represents the fitting parameter, which is a negative value.

As the spectral shift is proportional to the RI variation of MF within the light path, the monitored wavelength shift with the incident power and viscosity of carrier liquid can similarly be expressed as

3 $$\lambda (P,\,\eta )=[{\lambda }_{0}-{\lambda }_{s}][\coth (\sigma \frac{P}{\eta })-\frac{\eta }{\sigma P}]+{\lambda }_{0}(P > 0),$$

where *λ*_0_ is the monitored initial wavelength (without incident power), *λ*_*s*_ is the corresponding monitored saturated wavelength at sufficient high incident power.

The experimental data for the water-based-MF-filled structure shown in Fig. 5 and those for the oil-based-MF-filled structure shown in Fig. 7 are fitted with Eq. (3). The results shown in Figs 5 and 7 indicate that the proposed theoretical model is in a good agreement with the experiments.

### Influence of temperature and external magnetic field

**Figure 8:**
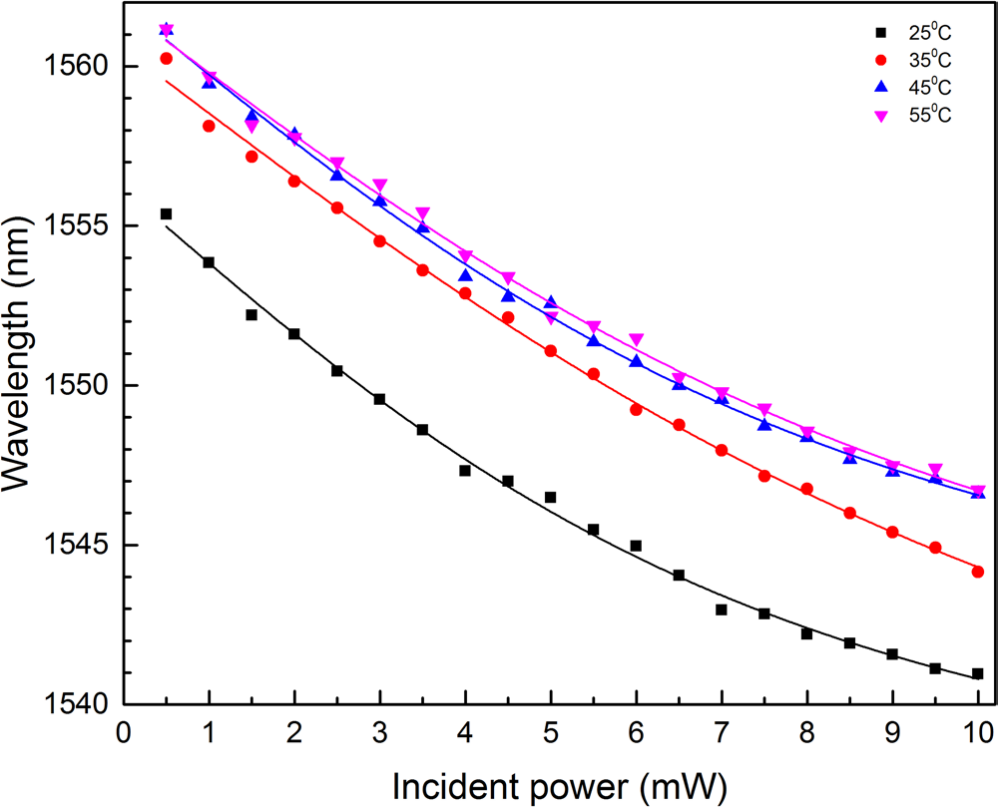
Spectral shift for the MF-FPI at different ambient temperatures.

The experimental results for the water-based-MF-FPI at temperatures of 25 °C, 35 °C, 45 °C and 55 °C are shown in Fig. 8. The tracked wavelengths are the interference peak wavelength at around 1555.36 nm, 1560.24 nm, 1561.12 nm and 1561.16 nm, respectively. Figure 8 displays that the spectrum shifts towards short wavelength with the incident power for all the ambient temperatures and the degrees of wavelength shift are almost the same in the same range of incident power. This implies that optical force has almost the same influence for the all temperatures studied. While at certain fixed incident power, individual experiments indicate that the tracked peak wavelength increases with the ambient temperature. This may be assigned to the increased thermal motion exerting on the MNPs with the increased ambient temperature^45,46,48^ and the thermal expansion of the fabricated structure. The thermal motion will disarrange the optical-force-induced outward movement of MNPs to a certain extent. Generally, the higher the ambient temperature is, the stronger the thermal agitation will be. Therefore, the thermal-induced hindrance of MNP’s outward movement is larger at higher ambient temperature. This has the “effective” effect of decreasing the incident power. So, the spectrum shifts towards long wavelength with the ambient temperature at certain fixed incident power.

**Figure 9:**
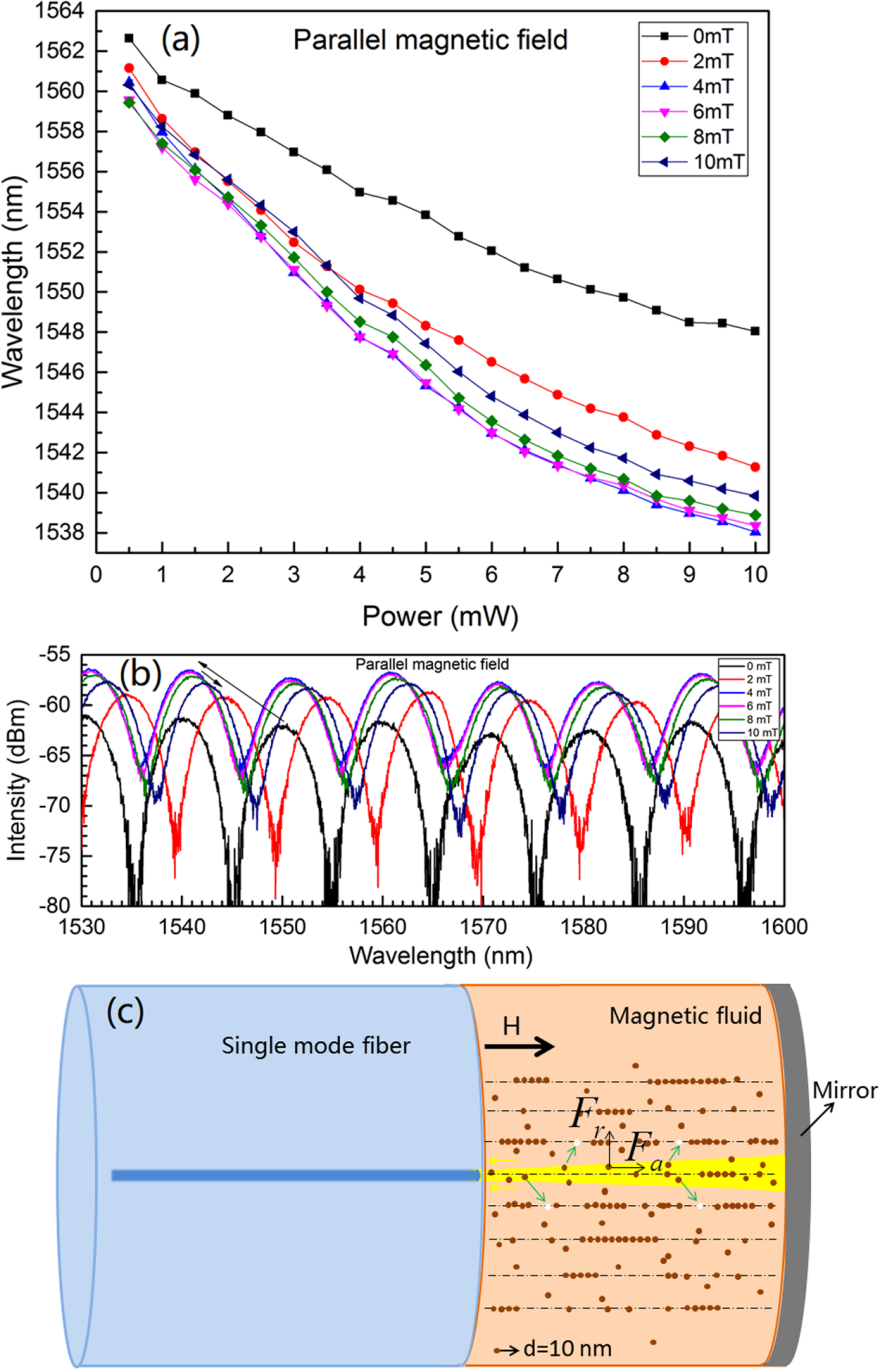
Spectral shift with incident power at different parallel magnetic field strengths (**a**), typical interference spectra at different magnetic field strengths while the incident power is 7.5 mW (**b**) and schematic diagram for the MNPs aggregation and optical-force-induced movement of MNPs (**c**).

**Figure 10:**
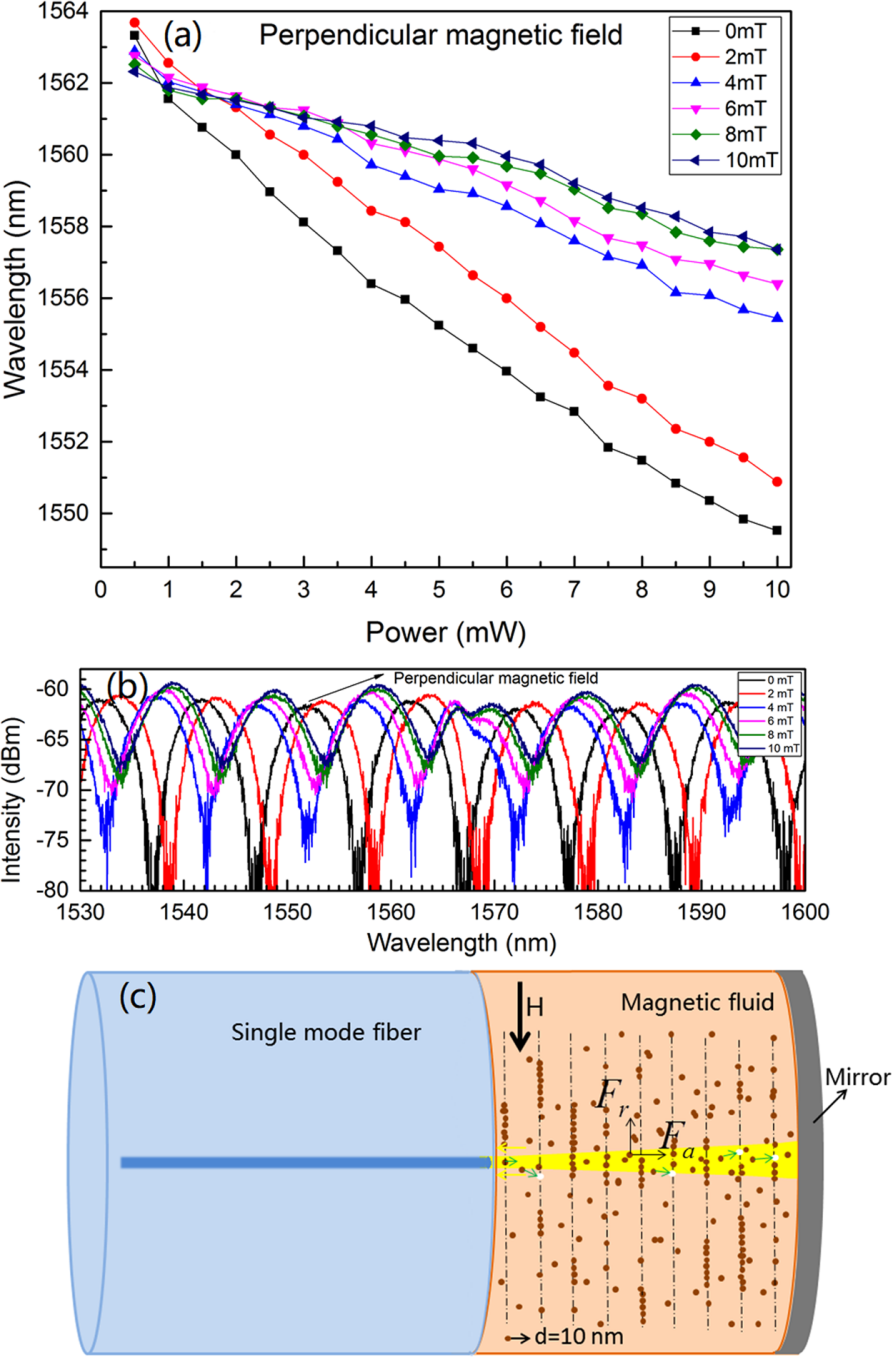
Spectral shift with incident power at different perpendicular magnetic field strengths (**a**), typical interference spectra at different magnetic field strengths while the incident power is 7.5 mW (**b**) and schematic diagram for the MNPs aggregation and optical-force-induced movement of MNPs (**c**).

Under external magnetic field, magnetic force will influence the MNPs besides optical force^22,23^. To investigate the influence of magnetic field strength and orientation on the optical-force-induced movement of MNPs, the parallel and perpendicular magnetic field (relative to the MF-FPI center axis) are applied, respectively (as shown in Figs 9(c) and 10(c)). The magnetic field strengths are set at 0 mT, 2 mT, 4 mT, 6 mT, 8 mT and 10 mT, respectively. The experimental results for the parallel magnetic field case are shown in Fig. 9(a), which implies that the degree of spectral shift increases with the magnetic field until ~5 mT at certain incident power. With further increase of magnetic field, the degree of spectral shift decreases. Figure 9(b) shows the typical interference spectra under different magnetic field strengths while the incident power is kept at 7.5 mW. In this configuration, the MNPs within the MF are aligned along the center axis as shown in Fig. 9(c). At low field regime, this ordered structuring is favorable for “magnifying” the repulsive optical force on MNPs along the perpendicular direction, which results in the larger RI variation in the light path for the relatively stronger magnetic field. But at high field regime, the magnetic attraction is very high and will restrain the optical-force-induced outward movement of MNPs. This leads to the backward shift of spectra with the magnetic field at the high field regime.

The experimental results for the perpendicular magnetic field case are shown in Fig. 10(a). The monotonic spectral shift with magnetic field is observed, which is different from that of the parallel magnetic field case. The typical interference spectra under different magnetic field strengths while the incident power is kept at 7.5 mW are shown in Fig. 10(b). The monotonic shift of interference spectra with the magnetic field is obvious. In this configuration, the MNPs will be aligned along the perpendicular direction as schematically shown in Fig. 10(c). The strong attraction between MNPs along the perpendicular direction hinders the optical-force-induced outward movement of MNPs. Then, the stronger the magnetic field is, the smaller the RI variation is. Hence, the degree of spectral shift decreases with the magnetic field as shown in Fig. 10(a).

**Figure 11:**
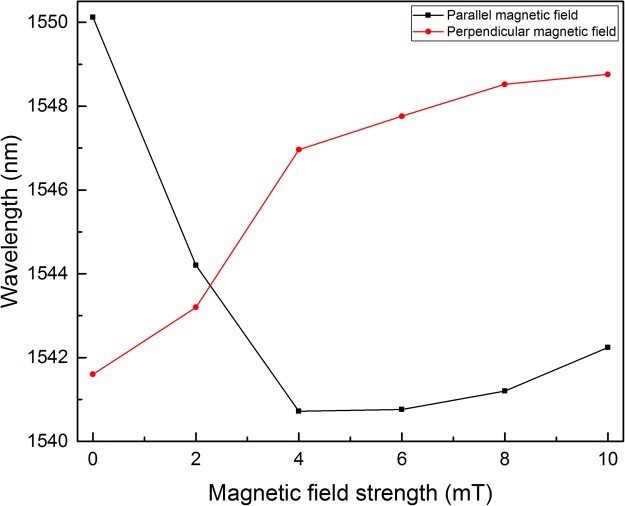
Peak wavelength shift with magnetic field strength at parallel and perpendicular magnetic field configurations. The incident power is 7.5 mW.

To explicitly reveals the influence of magnetic field, the magnetic-field-dependent shift for the tracked peak wavelength are replotted in Fig. 11 according to Figs 9 and 10. The incident power is set at 7.5 mW. Figure 11 implies that the influence of magnetic field strength and orientation on the optical response of the structure is remarkable. Moreover, the results for the parallel and perpendicular configurations are quite different. These further prove the optical force effect on the MNPs within the MF.

## Conclusion

The repulsive behavior for the optical force acting on the MNPs is investigated with MF-FPIs. The local MF’s RI variation in the light path is assigned to the optical-force-induced MNP’s concentration change. The influence of MF’s viscosity, ambient temperature and external magnetic field on the optical-force-induced MNP’s movement are experimentalized in details. The MF’s viscosity influences the optical-force-driven MNP’s movement remarkably. Increasing ambient temperature has the “effective” effect of decreasing the incident power. The applied parallel magnetic field is found to be favorable for “magnifying” the repulsive optical force on the MNPs under low field strength regime (0–5 mT), but further higher field (5–10 mT) will “weaken” the optical force effect. On the other hand, the perpendicular magnetic field inhibits the repulsive behavior of optical force monotonously. The corresponding micro-processes and physical mechanisms are analyzed and clarified. The results of this work may have the potential applications in light-controllable photonic devices and vector magnetic field detection.

## Methods

### Fabrication of the MF-FPI

A piece of coating-stripped SMF with flat-cleaved end is inserted into a capillary with the help of a microscope. The inner diameter, outer diameter and length of the capillary are 126 μm, 3 mm and 20 mm, respectively. The water-based and oil-based MFs are supplied by Beijing Sunrise Ferrofluid Technological Co., Ltd. and Ferrotec Corporation (Japan), which are employed to fill the capillary from the open end. The reflector is then fixed on the open end of the capillary. The UV glue is used to seal the MF-filled capillary. The obtained cavity lengths of the as-fabricated MF-FPIs are 92.9 μm and 51.5 μm, respectively.

## Electronic supplementary material

Supplementary Video
